# Molecular archaeoparasitology identifies cultural changes in the Medieval Hanseatic trading centre of Lübeck

**DOI:** 10.1098/rspb.2018.0991

**Published:** 2018-10-03

**Authors:** Patrik G. Flammer, Simon Dellicour, Stephen G. Preston, Dirk Rieger, Sylvia Warren, Cedric K. W. Tan, Rebecca Nicholson, Renáta Přichystalová, Niels Bleicher, Joachim Wahl, Nuno R. Faria, Oliver G. Pybus, Mark Pollard, Adrian L. Smith

**Affiliations:** 1 Department of Zoology, Peter Medawar Building for Pathogen Research, University of Oxford, Oxford OX1 3SY, UK; 2 Research Laboratory for Archaeology and the History of Art, University of Oxford, Oxford OX1 3QY, UK; 3 Rega Institute for Medical Research, Clinical and Epidemiological Virology, Department of Microbiology and Immunology, KU Leuven-University of Leuven, 3000 Leuven, Belgium; 4 Archäologie und Denkmalpflege der Hansestadt Lübeck, 23566 Lübeck, Germany; 5 Oxford Archaeology Ltd., Janus House, Osney Mead, Oxford OX2 0ES, UK; 6 Masaryk University Brno, 60177 Brno, Czech Republic; 7 Hochbauamt der Stadt Zürich, Abteilung Unterwasserarchäologie, 8008 Zürich, Switzerland; 8 Universität Tübingen, 72070 Tübingen, Germany; 9 Regierungspräsidium Stuttgart, Landesamt für Denkmalpflege, 78467 Konstanz, Germany

**Keywords:** ancient DNA, genetics, parasitology, archaeology, diet, trade

## Abstract

Throughout history, humans have been afflicted by parasitic worms, and eggs are readily detected in archaeological deposits. This study integrated parasitological and ancient DNA methods with a large sample set dating between Neolithic and Early Modern periods to explore the utility of molecular archaeoparasitology as a new approach to study the past. Molecular analyses provided unequivocal species-level parasite identification and revealed location-specific epidemiological signatures. Faecal–oral transmitted nematodes (*Ascaris lumbricoides* and *Trichuris trichiura*) were ubiquitous across time and space. By contrast, high numbers of food-associated cestodes (*Diphyllobothrium latum* and *Taenia saginata*) were restricted to medieval Lübeck. The presence of these cestodes and changes in their prevalence at approximately 1300 CE indicate substantial alterations in diet or parasite availability. *Trichuris trichiura* ITS-1 sequences grouped into two clades; one ubiquitous and one restricted to medieval Lübeck and Bristol. The high sequence diversity of *T.t*.ITS-1 detected in Lübeck is consistent with its importance as a Hanseatic trading centre. Collectively, these results introduce molecular archaeoparasitology as an artefact-independent source of historical evidence.

## 1. Introduction

Enteric parasites have afflicted humans throughout history and remain common in parts of the developing world. Indeed, prior to improvements in hygiene and medicine in the eighteenth century, enteric parasites were prevalent throughout the world, which makes them an attractive target for investigation of historical events. Previous molecular studies of historical infections have largely focused on high-impact diseases such as plague, leprosy, smallpox, malaria, and tuberculosis [1–5]. These studies typically focus on one, or very few, samples from a site and are targeted largely towards the identification of the pathogen under investigation [3,6–12]. Highly pathogenic infectious diseases often cause acute disease, interfere with the daily lives of infected individuals (in extreme cases killing them), and can be difficult to detect in archaeological contexts if identification relies on analysis of mummified or skeletal remains.

By contrast, enteric worms (helminths) do not have devastating clinical effects and the eggs are readily detectable in a wide variety of archaeological contexts associated with human faecal material [13–16]. Helminth is a collective term encompassing nematodes (roundworms), trematodes (flatworms), and cestodes (tapeworms), and there are many examples from each group that infect a range of hosts, including humans. Helminths exhibit wide variation in their life cycles: for example, *Trichuris trichiura* and *Ascaris* spp. (both nematodes) are transmitted via the faecal–oral route whereas the cestodes (tapeworms) *Taenia* spp. and *Diphyllobothrium latum* enter humans by consumption of undercooked red meat or freshwater fish, respectively. Hence, the type and number of eggs found in a deposit can be used to interpret levels of hygiene and/or dietary habits. In our experience, greater than 95% of pre-eighteenth century latrine and other communal deposit samples contain helminth eggs. The high prevalence of enteric helminths, their low pathogenicity, and the robustness of eggs encapsulating contained ancient DNA (aDNA), are features that underpin their broad applicability to historical and archaeological questions. A small number of studies of parasites in archaeological contexts have reported the use of aDNA as a diagnostic tool. These include the detection of protists in preserved tissue samples such as *Trypanosoma cruzi* from Peru, Northern Chile and Brazil or *Plasmodium falciparum* in Egyptian mummies and a fifth century CE Roman infant [17–20]. In terms of intestinal helminth infections, PCR detection and sequence data have been reported for *Trichuris trichiura*, *Ascaris* spp., *Taenia* spp., *Diphyllobothrium latum*, *Fasciola hepatica*, *Clonorchis sinensis*, *Schistosoma* spp., and *Enterobius vermicularis* [15,16,21–24]. However, in all of these cases, analyses were focused on diagnosis and often involved only a single archaeological site. Here, we employ molecular methodologies for species-level diagnosis and identification of epidemiological patterns by integrating data from multiple sites.

We analysed the occurrence of helminth parasite eggs within 152 samples that dated between Neolithic (*ca* 3600 BCE) and the Post Medieval (seventeenth century) which provided insight into historical living conditions and cultural practices. Parasite aDNA was amplified and sequenced to provide species-level diagnosis and, with *Trichuris trichiura* (the most frequently detected parasite), epidemiological patterns including genetic diversity provided a unique, artefact-independent source of historical information. The novel approaches developed in this work demonstrate the wide applicability and potential for molecular archaeoparasitology to broadly impact the study of historical events ranging from health to socio-economic and dietary practices.

## 2. Results

### (a) Site and sample description

The current study examined 152 samples from six sites in the UK, Germany, the Czech Republic, and Switzerland dating between Neolithic and Post Medieval periods (electronic supplementary material, figure S1) which included a large structured sample set from Medieval Lübeck. This city was one of the most influential trading cities in Medieval Europe and the founding centre of the Hanseatic League. The samples from Lübeck were obtained from the recent UNESCO excavation in the Gründungsviertel (founding quarter) between Fischstrasse and Alfstrasse, consisting of 31 stratigraphed latrine samples from eight houses dating between twelfth and seventeenth century CE. Further samples obtained for this study include a set from a medieval communal waste ditch in Bristol (UK, 26 samples), a Neolithic stilt settlement in Zurich (CH, 15 samples [25]), a Viking-age latrine in York (UK, one sample), and samples from the intestinal area of single burials in Břeclav-Pohansko (CZ, 61 early medieval samples) and Ellwangen-Jagst (DE, 28 medieval to early modern samples). The excavating archaeologists for all sites reported good to excellent morphological preservation of organic material in the archaeological deposits.

### (b) Microscopic detection of helminth parasites

**Figure 1.**
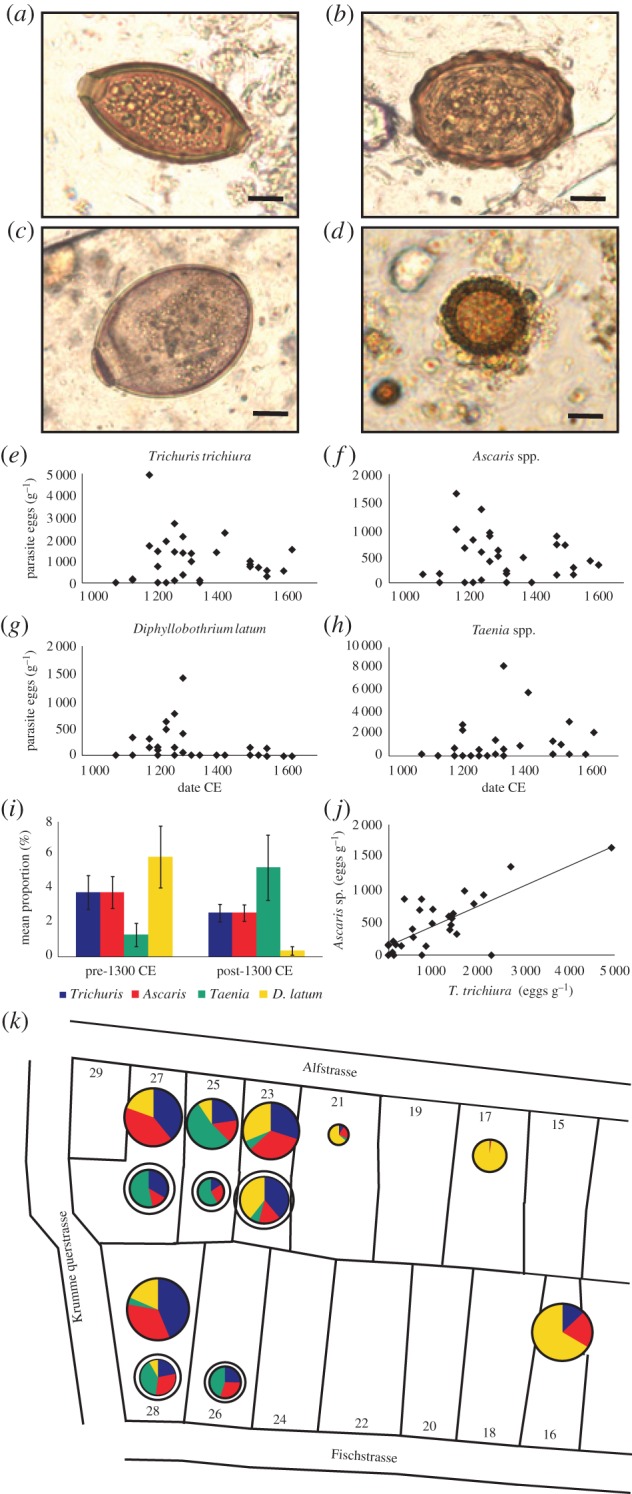
Identification and enumeration of helminth eggs in Lübeck deposits reveal a temporal pattern of cestode infections. Micrographs of parasite eggs in archaeological samples from Lübeck (*a*–*d*), images of eggs in Lübeck samples are representative of those detected in other sites. *Trichuris* spp. (*a*), *Ascaris* spp. (*b*), *Diphyllobothrium* spp. (*c*), and *Taenia* spp. (*d*) Scale bar: 10 µm. Numbers of parasite eggs in Lübeck samples (*e*–*h*) by species over time; *Trichuris* spp. (*e*), *Ascaris* spp. (*f*), *Diphyllobothrium* spp. (*g*), and *Taenia* spp. (*h*). Each point represents a sample. Summary of numbers of parasites of different species pre-post-1300 CE, mean % ± s.e.m. (*i*). The numbers of *Trichuris* spp. and *Ascaris* sp. eggs/g in each sample are correlated (*j*). (*k*) A detailed map depicting the plots sampled within Lübeck indicating the proportion of each parasite (pie charts) in each household. The upper, un-outlined pie charts represent samples dating prior to 1300 CE and the lower white outlined pie charts represent samples dated post-1300 CE. The segment area depicts the proportion of each parasite in each location and the overall size of the pie chart is scaled to the total number of parasites detected.

Helminth eggs are robust and display morphological characters that allow genus-level identification of parasites in archaeological deposits. The eggs of parasitic nematodes, *Ascaris* spp. and *Trichuris* spp., were detected in samples from all sites, and 94.5% of latrines or communal deposits (73 samples) were positive for eggs of at least one nematode. Cestode eggs were detected in samples from Neolithic Zurich, Viking York (*Taenia* spp.), and Medieval Lübeck (*Taenia* spp. and *Diphyllobothrium latum*). Representative micrographs of helminth eggs from Lübeck are depicted in figure 1; *Trichuris* spp. (figure 1*a*), *Ascaris* spp. (figure 1*b*), *Diphyllobothrium* spp. (figure 1*c*), and *Taenia* spp. (figure 1*d*). All 31 latrine samples obtained from Lübeck contained *Trichuris* and/or *Ascaris* eggs (ranges of 107–4 935/g, figure 1*e* and 45–1 645/g, figure 1*f*, respectively) and in 25 of 26 samples from the medieval port area of Bristol (Finzels Reach; ranges of 78–8 559/g and 76–1 162/g for *Trichuris* and *Ascaris*, respectively). The samples from Lübeck also contained substantial numbers of eggs from two cestode parasites, 14 of 31 positive for *D. latum* (range 49–1 414/g, figure 1*g*) and with 19 of 31 samples positive for *Taenia* spp. (range 133–8 310/g, figure 1*h*). Although *Taenia* spp. eggs were detected in samples from Neolithic Zurich and Viking York, these were too rare to allow a reliable estimation of density.

### (c) Molecular identification of parasites

**Figure 2.**
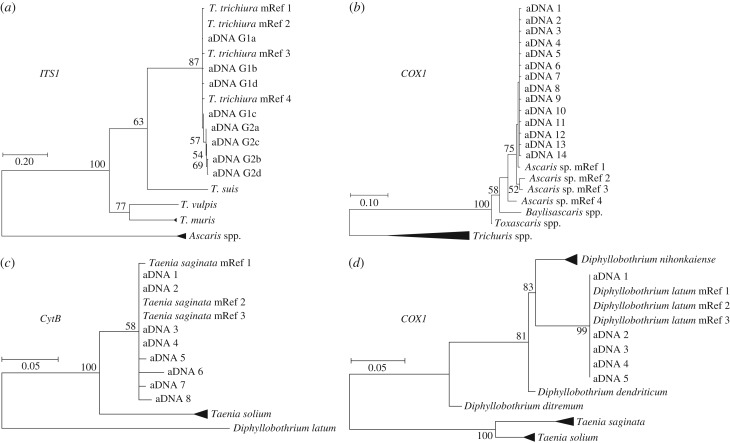
Molecular identification of helminth species in archaeological deposits. Maximum Likelihood Phylogenetic trees based on the Tamura–Nei model were calculated (1 000 bootstrap replicates, displayed as percentage score) to identify parasite species from aDNA PCR products. Modern reference sequences (mRef 1–4) from GenBank were clustered to 99% identity (see electronic supplementary material table S7 for ascension numbers). Sequences from species without aDNA sequences in each tree were collapsed. *Trichuris* spp. *ITS-1* tree (*a*) confirmed the identity of aDNA-derived sequences as *T. trichiura* when compared to other *Trichuris* spp. (*T. trichiura*, *T. muris*, *T. suis*, and *T. vulpis*) with *Ascaris* sp. as an outgroup. The aDNA provided 744 unique aDNA sequences (of 61 110 total) that formed eight clusters at 99% identity that fall into two groups (referred to as G1a-d and G2a-d). *Ascaris* sp. *COX1* tree (*b*) confirmed the identity of aDNA-derived sequences when compared to other Ascarididae (*Baylisascaris* sp. and *Toxascaris* sp.) or with *Trichuris* spp. (*T. trichiura*, *T. suis*, *T. discolour*, *T. ovis*, *T. vulpis*, *T. muris*, *T. skrjabini*, and *T. arivcolae*) as an outgroup. The aDNA provided 168 unique aDNA sequences (of 8 086 total) that formed 15 clusters at 99% identity (aDNA1–15). *Taenia* spp. *CytB* tree (*c*) confirmed the identity of aDNA-derived sequences as *T. saginata* rather than *T. solium* with *Diphyllobothrium latum* as an outgroup. Eight distinct aDNA sequences (from 15 in total) are represented (aDNA1–8). *D. latum* *COX1* tree (*d*) confirmed the species identity of aDNA-derived sequences when compared to other *Diphyllobothrium* spp. (*D. nihonkaiense*, *D. dendriticum*, and *D. ditremum*) employing *T. saginata* and *T. solium* as outgroups. Five distinct aDNA sequences (from 40 in total) were obtained (aDNA1–5).

To confirm/identify microscopic diagnosis of parasite species, aDNA was extracted and a PCR-amplified fragment was sequenced for targets within *Trichuris* (*ITS-1* and *β-tubulin*, figure 2*a* for *ITS-1*), *Ascaris* (*CytB* and *COX1*, figure 2*b* for *COX1*), *Taenia* (*CytB*, figure 2*c*), and *Diphyllobothrium* (*COX1*, figure 2*d*). Putative parasite sequences were identified using a pipeline involving BLAST (against the NCBI GenBank database) with their identity confirmed by constructing maximum-likelihood phylogenies (figure 2). The Transition/Transversion (Ts : Tv) ratios for aDNA targets were greater than those from modern sequences (electronic supplementary material, tables S1 and S2). However, with *Trichuris,* although the TsA:TsB ratio for Lübeck and York was higher than that for modern samples it was lower for other sites indicating non-aDNA damage-associated variation. For *Trichuris*, the sequence data obtained from aDNA grouped with the human parasite *T. trichiura* and were distinct from other *Trichuris* spp., including those typically found in pigs, dogs, and rodents (*T. suis*, *T. vulpis*, and *T. muris*, respectively; representative data depicted in figure 2*a*). Sequence analysis of PCR products targeting *Taenia* (*CytB*, figure 2*c*) and *Diphyllobothrium* (*COX1*, figure 2*d*) from Lübeck samples identified these as *T. saginata* and *D. latum*, respectively.

### (d) Temporal distribution of helminth eggs

As the two nematode parasites *Trichuris* and *Ascaris* were commonly found, we evaluated the correlation between the numbers of these two parasites in Lübeck and Bristol using separate Generalized Linear Mixed Models (GLMMs). A strong positive correlation between the numbers of the two parasites were found in Lübeck (figure 1*j*, estimate ± s.e.: 0.31 ± 0.07, 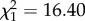, *p* < 0.001) and Bristol (electronic supplementary material, figure S2, *ρ* = 0.590, *S* = 1 200.00, *p* = 0.00153).

Lübeck was the only location where cestode eggs were detected consistently throughout the sampling period. Despite both cestode species being present at high numbers in Lübeck their occurrence was largely non-overlapping, with only five samples containing eggs from both parasites. The prevalence of *Diphyllobothrium* and *Taenia* eggs showed a clear time dependency (figure 1*g*,*h*; GLMM with Poisson error distribution model, *Diphyllobothrium* estimate ± s.e.: −3.15 ± 1.26, 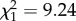, *p* = 0.0023 and *Taenia* estimate ± s.e.: 3.34 ± 1.17, 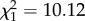, *p* = 0.0015) with *D. latum* more prevalent in earlier samples and *Taenia* in later samples. To assess whether these shifts are best explained by a gradual change over time or by a sudden shift in parasite prevalence, we used a model using the date as a predictor of parasite status. Bootstrapping strongly supports a structural break in the data (greater than 99% of bootstrap replicates for *D. latum*, and 96% for *Taenia*). For *D. latum*, the break most likely occurs between 1300 and 1325 CE (92% of replicates, with other possible breakpoints each suggested by less than 1.5% of replicates). For *Taenia*, the breakpoint was slightly less abrupt, with a most likely timing of between 1300 and 1325 CE (60% of replicates) or between 1350 and 1400 CE (35% of replicates). A binomial test gives a *p*-value of less than 0.001 for the 1300/1325 CE breakpoint for *D. latum*, and *p*-values of less than 0.001 for either of the possible breakpoints for *Taenia* sp. These changes in parasite occurrence and abundance across the *ca* 1 300 CE boundary were evident only for the cestode parasites. We analysed variation in parasite occurrence for Lübeck using GLMMs with binomial error distribution. For each parasite, we entered separate GLMMs using various factors to assess the correlations. All models were checked for over- or under-dispersion. In Poisson-distributed data, we added an observation-level random factor in the model whenever over-dispersion was detected [26,27]. No under- or over-dispersion was detected for binomially distributed data. Because Bristol had only four samples post-1300 CE, we used non-parametric tests (Fisher's exact Mann–Whitney test). The occurrence and count of the nematodes were independent of time in Lübeck (occurrence *Ascaris* estimate ± s.e.: 0.77 ± 0.95, 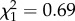, *p* = 0.41, counts *Ascaris* estimate ± s.e.: −0.71 ± 0.42, 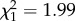, *p* = 0.16; occurrence *Trichuris* estimate ± s.e.: 0.39 ± 1.04, 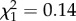, *p* = 0.71, counts *Trichuris* estimate ± s.e.: −0.66 ± 0.37, 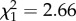, *p* = 0.10, figure 1*e,f*,*i*) and Bristol (occurrence *Ascaris*: odds ratio 0.68, *p* = 1.00, counts *Ascaris* Mann–Whitney *p* = 0.36; occurrence *Trichuris*: odds ratio 0.16, *p* = 0.29, counts *Trichuris* Mann–Whitney *p* = 0.15).

When considering the location of samples within the Lübeck excavation (i.e. house and street location; figure 1*k*) all seven houses with pre-1300 CE samples contained *Trichuris*, *Ascaris*, and *D. latum* eggs and four contained low levels of *Taenia* spp. (except for Alfstrasse 25 which contained larger numbers of *Taenia* spp. eggs). Alfstrasse 17 and 21 contained fewer *Trichuris* and *Ascaris* eggs than other contemporary samples, but the overall parasite load was especially low in Alfstrasse 17 (figure 1*k*). *Taenia*, *Trichuris*, and *Ascaris* eggs were present in all locations where samples were obtained post-1300 CE. Notably, *D. latum* was detected in post-1300 CE samples from two houses (Alfstrasse 23 and Fischstrasse 26) although only Fischstrasse 26 contained high numbers of eggs.

### (e) The source of cestode infections in Lübeck

**Figure 3.**
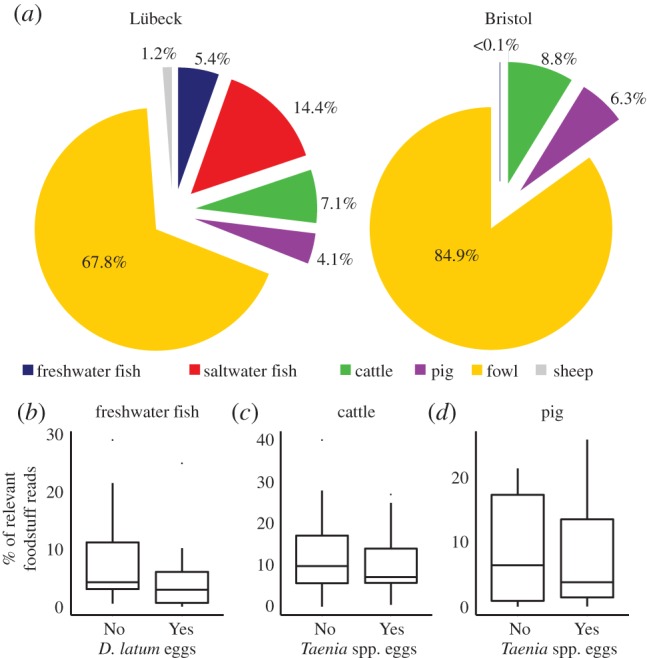
Detection of potential food animal species aDNA to identify candidate sources of cestode infections. Potential food animal species were identified using a mitochondrial 16S-rDNA targeting PCR. Outputs derived from Lübeck and Bristol were grouped into freshwater fish, saltwater fish, cattle, pigs, fowl, and sheep/goat (*a*). Note that there were different patterns detected in the two sites, including the proportion of fresh and saltwater fish. A full description of the species identified is given in electronic supplementary material, table S3. Box and whisker plots indicate the median (line) with the first and third quartile (box) and one standard deviation (whisker) comparing the proportion of relevant food aDNA in *Diphyllobothrium latum* or *Taenia* spp. egg positive and negative samples from Lübeck. The proportion of freshwater fish aDNA signal in samples with and without *D. latum* eggs (*b*), proportion of cattle (*c*) and pig (*d*) aDNA signal in samples with and without *Taenia* spp. eggs. There were no significant differences according to the proportion (or presence/absence) of any food-species-derived aDNA in samples containing cestode eggs, or not containing eggs.

The sample set from medieval Lübeck provided a unique opportunity to investigate aspects of diet, as the prevalence of *D. latum* and *Taenia* spp. indicates the widespread consumption of contaminated raw or undercooked freshwater fish (*D. latum*) and red meat (pork for *T. solium* or beef for *T. saginata*). The changing prevalence of these two parasites may have resulted from changes in consumption of contaminated freshwater fish or red meat. A wide range of freshwater fish species could be the source of *D. latum*, and the change in prevalence at around 1300 CE might be due to changes in the consumption of particular freshwater fish species. Previous studies have successfully amplified genetic material of plants and animals from archaeological sediments and used these to identify resident species [28,29]. We employed PCR amplification of a short fragment of the eukaryotic mitochondrial 16S gene and sequencing (MiSeq) to identify putative food species [30]. Sequences matched a range of targets, including domesticated and wild animals as well as humans. The most common sequences were human, followed by a range of potential food species including cattle (*Bos taurus*), pigs (*Sus scrofa*), sheep and goat (*Caprinae*), fowl (*Galloanserans*) as well as various freshwater and marine fish species. Although extreme care must be exercised when interpreting the vertebrate aDNA food signature, the patterns obtained in samples from Lübeck were distinct to those detected in any other site, including Bristol (figure 3*a*). As an additional control, we also included DNA extracted from six sub-surface (approx. 20 cm deep) soil samples from locations within Oxford which did not yield any food or human-related sequences. Although the aDNA food signature can only be used as a semi-quantitative measure, the composition and estimated proportions of food-related sequences identified within this study of Lübeck were comparable with osteological analyses from previous excavations [31–35]. Cod (*Gadus morhua*) and herring (*Clupea haerengis*) were the most prevalent fish sequences detected in Lübeck, but were not detected in other sites. This was not unexpected because cod and herring were major bulk trading goods of the Hanseatic League (air-dried or salted). The Lübeck samples also contained aDNA that identified a wide range of freshwater fish as potential sources of *D. latum* infection, including cyprinids, coregonids, perch, pike, and eel. The most straightforward explanation would be a relative decline in freshwater fish consumption. However, neither the occurrence nor prevalence of all freshwater fish or any individual fish species changed dramatically across the *ca* 1 300 CE boundary after which *D. latum* largely disappeared from the Lübeck samples. Furthermore, there was no correlation between *D. latum* prevalence and the proportion or type of freshwater fish aDNA (figure 3*b*; electronic supplementary material, table S3). In Lübeck, the most common freshwater fish family by sequence frequency were the Cyprinidae that could be subdivided into two subfamilies (*Cyprininae* and *Leuciscinae*). When considering the number of samples where each freshwater fish was detected, the most frequently detected were *Coregonus* spp. (17/31 samples) and *Leuciscinae* (16/31 samples). Both coregonids and cyprinids are candidate sources of *D. latum* infection in humans although it is possible that multiple fish species were involved in the transmission cycle. *Taenia saginata* was identified in Lübeck samples indicating a definite source of infection from contaminated beef. Cattle and pig sequences were identified in the majority of samples (30/31 and 25/31, respectively) but in neither case did the magnitude of bovine or porcine signal (proportion of total food-related reads) associate with the prevalence or number of *Taenia* eggs (figure 3*c*,*d*).

### (f) Genetic analysis of *Trichuris trichiura* aDNA

**Figure 4.**
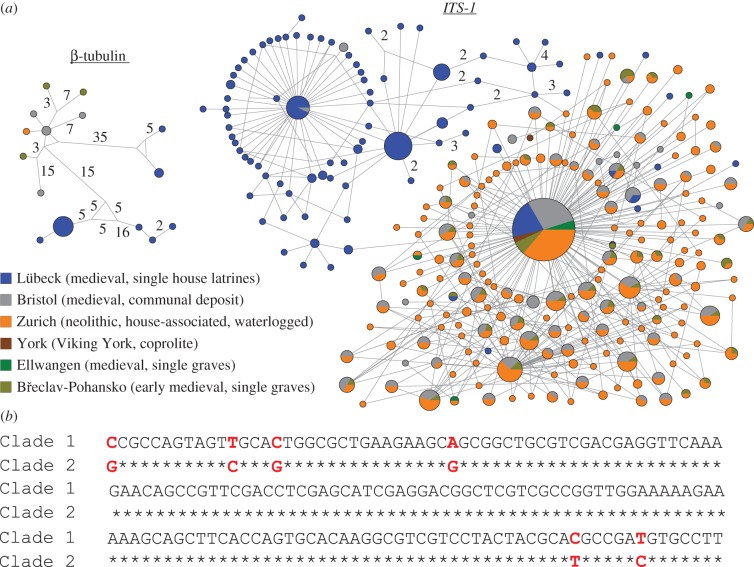
Haplotype networks of *T. trichiura* *ITS-1* and β-tubulin. (*a*) The haplotype network displays the distance between two sequences. Each node represents a haplotype, of which some are shared between locations, and each edge represents a single change in the sequence (unless otherwise indicated). The node size represents the number of sequences within the node (identical sequences) and the colouring indicates the site from which the sequence was generated. If a sequence occurs in more than one site, the proportion is represented by fractional colouring of the node. The network clearly indicates a divide between the majority of the ancient sequences and a group of sequences from Lübeck and Bristol. (*b*) The alignment of consensus sequences of groups 1 and 2 of *T. trichiura* *ITS-1* reveals six distinct nucleotide changes which define the identity of the groups.

*ITS-1* sequences have previously been used for species identification of *Trichuris* [36]. As *T. trichiura* was present in a wide range of sites, we used *ITS-1* (744 unique aDNA sequences, identical replicates were clustered) and *β-tubulin* (34 unique aDNA sequences) sequence alignments to analyse and compare *T. trichiura* populations in the different sampling sites. First, median-joining haplotype networks were inferred for the *ITS-1* and *β-tubulin* (figure 4*a*) alignments, using NETWORK 4.6.6 (fluxus-engineering.com, [37]) with *ɛ* = 0. The haplotype distributions for *T. trichiura ITS-1* (TtITS-1) comprised two large groups of sequences, one that contained linked sequences from all sites (comprising of 46 547 aDNA sequences; this group would include all modern reference sequences, but these were omitted from the analysis) and a second smaller group (comprising of 14 563 aDNA sequences) that was dominated by haplotypes present in the Lübeck site, which also contained a small number of haplotypes from Medieval Bristol (figure 4*a*). The Lübeck-dominated group 2 can be segregated from group 1 by virtue of six conserved nucleotide polymorphisms (figure 4*b*). Although care should be exercised when identifying a novel haplotype based upon aDNA data, the high frequency and nature of the haplotype sequence (six conserved polymorphisms with only one C-T transition and no G-A transitions) indicate that it is highly unlikely that this haplotype group was derived from aDNA-associated damage. Despite the much smaller number of sequences available for *T. trichiura β-tubulin*, these also display two groups with one dominated by sequences only found in Lübeck (figure 4*a*). Hence, Lübeck appears to be associated with a different pattern of genetic variability than the five other locations and a subset of the sequences from Lübeck forms a distinct cluster within the haplotype network. For TtITS-1, sub-networks were constructed to assess spatial or temporal patterns. Only location-associated patterns were observed and no patterns were evident when sub-networks were generated for shorter time periods confirming that the two groups were present in Lübeck throughout the sampling period (electronic supplementary material, figure S3a,b). Some samples were dominated (greater than 99%) with either group 1 or 2 TtITS-1 sequences while others included mixed populations. Intriguingly, while samples with relatively few eggs (less than 500) contained mixed populations (5/6 samples) those that contained larger numbers of eggs were dominated (greater than 99%) by a single group (5/9 with group 2 and 4/9 with group 1 sequences).

Lübeck contained the greatest nucleotide diversity [38] of TtITS-1 sequences; almost two times higher than in Bristol, Zurich, and Břeclav-Pohansko, and around four times higher than in York and Ellwangen. Furthermore, Lübeck was the only location with a relative nucleotide diversity [39] greater than one and this was at least three times higher than detected for the other locations (electronic supplementary material, table S4). Regarding genetic differentiation among locations, estimates of pairwise *Φ*_ST_ and associated significance tests revealed that Lübeck as the only location which is significantly differentiated from the other locations (electronic supplementary material, table S4). Only Lübeck and Bristol had a significant spatial structure, as measured by the Association Index statistic (*p*-value < 0.001). The maximum monophyletic clade size for Lübeck (41.590) was also much higher than that measured for Bristol (5.446, electronic supplementary material, table S4). It is important to note that these diversity measures are both corrected for unequal sample size, which makes possible their comparison among populations/sampling locations for which we have different sample sizes.

## 3. Discussion

The high occurrence of infection, low pathogenicity, and robust nature of the eggs from intestinal helminths support their use in archaeological contexts, including aDNA studies. Here, we integrated parasitological and aDNA methodologies on parasite material derived from a variety of sites ranging from *ca* 5600 to 350 years old. All sites contained eggs from two nematode parasites *Trichuris* spp. and *Ascaris* spp., and the samples from Lübeck contained high numbers of eggs from two cestodes *D. latum* and *Taenia* spp. Sequences of amplified aDNA confirmed parasite identity and clearly established that they were of human origin. Parasite aDNA sequence was obtained from 75.9% of samples where eggs were identified microscopically, independent of soil condition (e.g. age or context), which indicates the broad applicability of the approach to a wide range of sample types. Parasite eggs are environmentally robust [40] and because intact eggs are likely to protect aDNA, it is not surprising that it is possible to amplify parasite aDNA by PCR from most samples.

The Medieval Hanseatic city of Lübeck had a unique parasitological character in terms of the numbers and temporal distribution of cestodes and the population genetics of the nematode *T. trichiura*. First, considering the cestodes, there was a clear temporal pattern for both *D. latum* and *Taenia* spp. eggs, with the former being present in most pre-1300 CE samples and in only two of the post-1300 CE samples. By contrast, *Taenia* spp. eggs increased in occurrence and prevalence in later samples. The overall numbers of *D. latum* and *Taenia* spp. eggs in Lübeck were much larger than reported in any archaeological study (e.g. [41]) which indicates that the sampled Medieval population of Lübeck engaged in food preparation practices that supported the acquisition of such infections (i.e. consumption of uncooked or undercooked fish and red meat).

The source of human *Taenia* infection was most likely from undercooked beef because the sequences that were obtained matched the beef tapeworm *T. saginata* rather than the pork tapeworm *T. solium*. The source of *D. latum* infection in the people of Medieval Lübeck will have been freshwater fish. Analyses of eukaryotic mitochondrial 16S sequences revealed a range of fish species that would be suitable second intermediate hosts for *D. latum*, either by consumption of infected first intermediate host (copepod) or other fish that had previously acquired the infection. The importance of local freshwater fisheries to Lübeck is supported by imperial charters dating from 1188 and 1226 awarding the city formal control of fishing rights over the local rivers and lakes [42,43].

It is important to consider the factors that may have led to the dramatic changes in the occurrence and prevalence of both cestode parasites. For *D. latum* there was a clear boundary *ca* 1300 CE, with most samples prior to this date containing *D. latum* eggs. The change in occurrence may have resulted from a change in dietary preferences, food preparation practices, or availability of contaminated freshwater fish. Some explanations are confounded by the detection of *D. latum* in two samples dated c1500 CE, although only one of these contained substantial numbers of parasites. The simplest explanation would be that less freshwater fish was consumed after 1300 CE. However, there was no detectable change in total freshwater fish or any single species of fish (by morphology or aDNA signature with the resolution provided here) that would explain the dramatic reduction of *D. latum*. With the expansion of the city around 1300 CE, the river west of Lübeck, the Wakenitz, became increasingly polluted by waste from the increased production of meat and leather [44] which could have affected the availability/attractiveness of fish from the Wakenitz, or numbers of copepods. The supply chain of freshwater fish could also have been affected by other factors including the conversion of the Benedictine monastery to a Cistercian nunnery and the relocation of the Benedictine monks about 50 km away to Cismar (1245–1247 CE) [43]. As the monastery had riverine fishing rights and stock ponds this change could have interrupted the availability of contaminated fish within the city. It is also possible that the availability of locally caught fish was affected by a shortage of fishermen following the Black Death (1346–1350) or by changes in trading patterns as Lübeck matured as an economic centre [33,45,46]. Although we cannot exclude any single factor affecting the incidence of *D. latum* the maintenance of freshwater fish aDNA signature across the 1300 CE boundary supports a hypothesis of a reduction in consumption of infected fish which is most easily explained by pollution reducing the numbers of infected copepods. The continued presence of *D. latum* in two households indicates that at least some contaminated fish remained available post-1300 CE, although at a much-reduced level. The temporal change in occurrence and prevalence of *T. saginata* was less pronounced but highly significant, suggesting an increase in the consumption of raw or undercooked beef from the mid-thirteenth century CE on. This change may reflect the increasing wealth of Lübeck's citizens or an increased supply of beef to the markets. In other medieval cities, the Black Death resulted in an increased focus on cattle due to shortages in manpower for arable farming which may have influenced the source of beef in Lübeck. The introduction of Rinderpest virus into Europe (early 1300s) which affected a large proportion of cattle [47] may also have affected the availability of infected beef. Whatever factors affected the prevalence of food-transmitted cestodes in Lübeck the magnitude of infection is without precedence and indicates the importance of local social/dietary practices affecting the prevalence of food-transmitted diseases.

The widespread incidence of faecal–orally transmitted nematode parasites (*Trichuris* and *Ascaris*), affords a novel opportunity to use molecular epidemiology to interrogate historical events. We used molecular approaches to identify parasite species and generated a considerable dataset of *T. trichiura* ITS-1 sequences to identify epidemiological signatures. The most diverse groups of TtITS-1 sequences were found in Lübeck and Bristol, both port sites where parasite diversity might be promoted by trade-based connectivity. Of these two locations, TtITS-1 sequences from Lübeck were substantially more diverse reflecting its high level of connectivity with other sites. Indeed, Lübeck was one of the most important ports in Medieval Europe controlling a considerable proportion of the trade in the Baltic Sea and was also the founding site and leading city of the Hanseatic League. We propose that the intra-specific parasite diversity seen in Lübeck was a consequence of extensive trade with, and unintentional import of parasites from, other parts of Europe. Intriguingly, Medieval Bristol contained the second most diverse population of TtITS-1 sequences, supporting the hypothesis that the connectivity associated with ports increased parasite diversity. Hence, parasite diversity may be useful as a marker for the level of interaction that any particular site has with other regions. A full examination of this hypothesis requires exploration of a wide range of sites but is supported by the fact that Bristol was a less prominent port (and contained less TtITS-1 diversity) than Lübeck and both of these contained more diversity than other non-port sites.

Phylogenetic analyses revealed two groups of TtITS-1 sequences. While group 1 sequences were ubiquitous, group 2 sequences were prominent within Lübeck, rare in Bristol, and absent from all other study sites and modern sequences. Despite a smaller number of sequences, a similar two clade pattern was observed for *T. trichiura β-tubulin* sequences with Lübeck represented in both groups. Hence, Lübeck contained a unique, genetically distinct parasite lineage that was circulating in the medieval period but is no longer present in modern *T. trichiura* populations. Importantly, the only non-Lübeck site where group 2 *T. trichiura* parasites were detected was Bristol, albeit at very low numbers. This distribution supports the premise that group 2 parasites were more likely to have been transferred to Bristol from Lübeck or that these ports were interacting very differently with an independent source location for group 2 parasites. Interestingly, within Lübeck group 1 and group 2 *T. trichiura* sequences were not evenly distributed although there were no temporal or spatial patterns. Further sequence data, including a much larger set of locations and DNA targets would be needed to confirm the nature and distribution of the, apparently extinct, group 2 lineage.

A combination of parasitological and molecular approaches was used to identify changes in eating habits and trading patterns centred on Lübeck, a dominant medieval port and founding city of the Hanseatic League. We propose that the integrated application of molecular parasitology and epidemiology to the study of archaeological sites represents a novel addition to the repertoire of approaches that can be used to interrogate historical events.

## 4. Methods

### (a) Sample handling and preparation workflow

aDNA handling practices have been outlined in a range of publications [48–50]. None of the parasites targeted in this study is endemic in the UK or any country where the material was received from. None of the laboratories has ever handled or stored modern samples containing these parasites, hence modern contamination is extremely unlikely. The processing pipeline involved sample preparation and PCR set up in a ‘clean’ laboratory within dedicated UV hoods using dedicated equipment with all handling of amplified material undertaken in a separate laboratory. Researchers also followed a strict unidirectional protocol, never entering the ‘clean area’ or handling samples after entering any downstream processing laboratory.

### (b) aDNA extraction

Soil subsamples (5 g) were re-hydrated in 20 ml of PCR-grade water (Qiagen, Hilden, Germany) as described above. The samples were then sieved through a series of disposable, single-use nylon mesh sieves with decreasing aperture size (1 030 µm, 500 µm, 100 µm; Plastok Associates Ltd, Birkenhead, UK). The eggs present in the filtrate were pelleted by centrifugation (400*g*, 10 min) and the pellet homogenized in a BeadBeater (BSP BioSpec, Bartlesville, USA) using 1 mm glass beads (Hecht, Sondheim, Germany). aDNA was extracted using a Qiagen Blood&Tissue or Mericon Food kit (Qiagen). Blank extraction controls were routinely included as contamination checks.

### (c) Microscopic diagnosis

Aliquots of the initial subsample were analysed microscopically using a Nikon Eclipse E400 with 10× and 40× lenses (Nikon UK, Kingston-Upon-Thames, UK). Photographs were recorded on a QImaging MP5.0 RTV camera (QImaging, Surrey, Canada). Parasite egg counts were extrapolated from replicated counts to the dry weight of the sample.

### (d) PCR amplification

The PCR was performed in two stages; with the first stage boosting the starting concentration using a very robust polymerase (AmpliTaq Gold 360, ThermoFisher, Loughborough, UK) and a second stage to produce sufficient amounts of target DNA using a high-fidelity polymerase and to attach the barcodes for MiSeq sequencing (Phusion Hot Start Flex, New England Biolabs, Hitchin, UK). PCR primers were designed based on published sequences or reported previously (electronic supplementary material, Methods). Multiple water controls were included with each set of PCRs, producing either no product or primer concatemers.

### (e) Sequencing

Second stage PCR products were cleaned up prior to sequencing (MinElute, Qiagen). Initial Sanger sequence data were generated using the BigDye Terminator (ThermoFisher).

Parallel sequencing libraries were made from pooled PCR products with barcoded primers. For the library prep NEBNext Ultra DNA Library Prep Kit for Illumina with NEBNext Multiplex Oligos for Illumina (NEB) were used. The libraries were sequenced on a MiSeq (Illumina, Chesterford, UK) using the MiSeq reagent kit v. 3 (600 cycle). Sequences were separated according to library barcode using Basespace (Illumina) and then processed using custom python scripts, which identified amplicons with the correct forward and reverse primers, and sorted according to sample barcode. Sample barcodes consisted of 7 bp attached to one primer (based on Bystrykh, [51]) with an edit (Hamming) distance of 2, enabling single error correction in the barcode. Sequencing was at the Wellcome Trust Centre for Human Genetics (MiSeq) and the Department of Zoology (MiSeq/Sanger), University of Oxford.

A total of 419 936 MiSeq reads were obtained from parasite PCR amplicons resulting in 129 494 total parasite-specific reads (electronic supplementary material, table S5). The pan-vertebrate 16S amplicons were sequenced to an overall depth of 13 460 879 with 1 768 632 vertebrate specific reads and 146 813 food-species-derived reads (electronic supplementary material, table S6).

### (f) Temporal distribution of helminth eggs

The correlations between the numbers of the two nematode parasites *Trichuris* and *Ascaris* in Lübeck and Bristol were evaluated using GLMMs. To assess whether these shifts are best explained by a gradual change over time or by a sudden shift in parasite prevalence, we used Akaike's information criterion [52].

### (g) Molecular phylogenetic analysis

Phylogenetic history was inferred by using the Maximum-Likelihood method under on the Tamura–Nei substitution model [53] using MEGA7 [54].

### (h) Haplotype networks

Median-joining networks were inferred for the *ITS1* and the additional *β-tubulin* gene fragments using NETWORK 4.6.6 (fluxus-engineering.com) [37] with *ɛ* = 0. For the haplotype network inferences, sequences including sequencing ambiguities were removed from the alignment and indels (insertion–deletion) were coded as substitutions. Haplotype networks inferred for *ITS1* and *β-tubulin* were separately coloured by sampling origin and by estimated dating. Furthermore, for *ITS1*, sub-networks were inferred for each time period with more than five identified sequences and coloured by sampling origin.

### (i) Genetic diversity and population structure

Nucleotide diversity and the relative nucleotide diversity were estimated for *ITS1* within each location using SPADS 1.0 [55]. We also used SPADS to estimate the pairwise *Φ*_ST_ statistics for *ITS1* [56] among the different locations.

## Supplementary Material

Supplementary Information

## Data accessibility

All ancient DNA sequences have been deposited on the NCBI GenBank, accessible under the accession numbers MH599138–MH600059.
