# Supplementary material for: Molecular archaeoparasitology identifies cultural changes in the Medieval Hanseatic trading centre of Lübeck
Source: Proc Biol Sci. 2018 Oct 3;285(1888):20180991. doi: 10.1098/rspb.2018.0991 (PMC6191690; doi:10.1098/rspb.2018.0991)
Supplement: Supplementary Information [file rspb20180991supp1.docx]

**Supplementary Information**

**Supplementary Figure S1: Locations and description of archaeological sites**


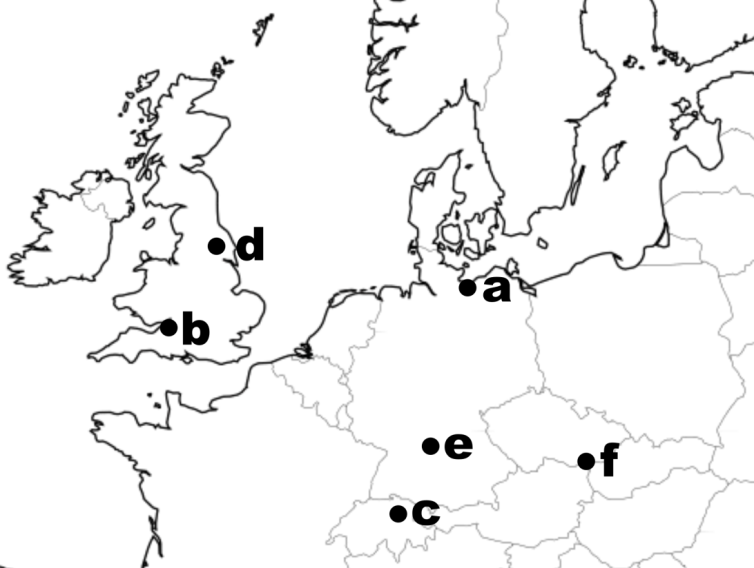


|  | Site Name and country | Context | Number of samples | Date range | Parasites detected | Number of microscopy positive samples | Number of sequence positive samples | Number of parasite aDNA sequences |
| --- | --- | --- | --- | --- | --- | --- | --- | --- |
| a | Gründungsviertel der Hansestadt Lübeck (DE) | latrine | 31 | c1100 CE to  c1650 CE | *Trichuris*  *Ascaris*  *Taenia*  *Diphyllobothrium* | 31 | 25 | 27,441 |
| b | Finzels Reach, Bristol (UK) | communal waste deposit | 26 | c1150 CE to  c1700 CE | *Trichuris*  *Ascaris* | 25 | 20 | 38,404 |
| c | Parking Opéra, Zurich (CH) | house-associated | 15 | 3176 BCE to  c3060 BCE | *Trichuris*  *Ascaris*  *Taenia* | 13 | 10 | 54,853 |
| d | Viking York  (UK) | coprolite | 1 | c1000 CE | *Trichuris*  *Ascaris*  *Taenia* | 1 | 1 | 162 |
| e | Ellwangen-Jagst  (DE) | single graves | 28 | c1400 CE to  c1600 CE | *Trichuris*  *Ascaris* | 12 | 9 | 74 |
| f | Břeclav-Pohansko (CZ) | single graves | 61 | c850 CE  to  c950 CE | *Trichuris*  *Ascaris* | 30 | 20 | 8,560 |

Map of sites from where samples were obtained with detailed sample descriptions including name of site, country of origin, archaeological context, number of samples, archaeological dating and genera of parasites detected, the number of samples positive for parasite eggs by microscopy and parasite sequences are given. Overall, 52.5% of samples were sequence positive representing 75.9% of those that were positive for parasite eggs by microscopy).

**Supplementary Figure S2: Correlation between the numbers of *Trichuris* and *Ascaris* eggs from the excavation of Finzels Reach, Bristol, UK**


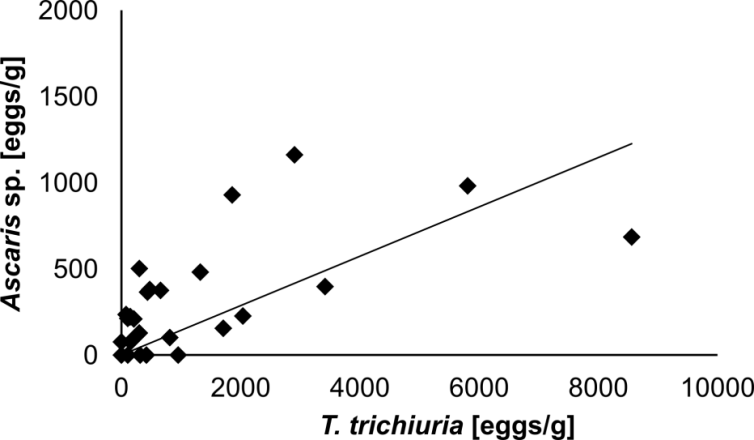


The presence of one of the two nematode parasites predicts the presence of the other one in the samples from the medieval port area of Bristol. Furthermore, the number of *T. trichiura* and *Ascaris* sp. eggs in a sample also correlate.

**Supplementary Figure S3:** **Haplotype networks of *Trichuris trichiura* ITS-1 and β-tubulin shaded (a) or divided (b) by time period indicates no temporal signature of the two sequence groups.**


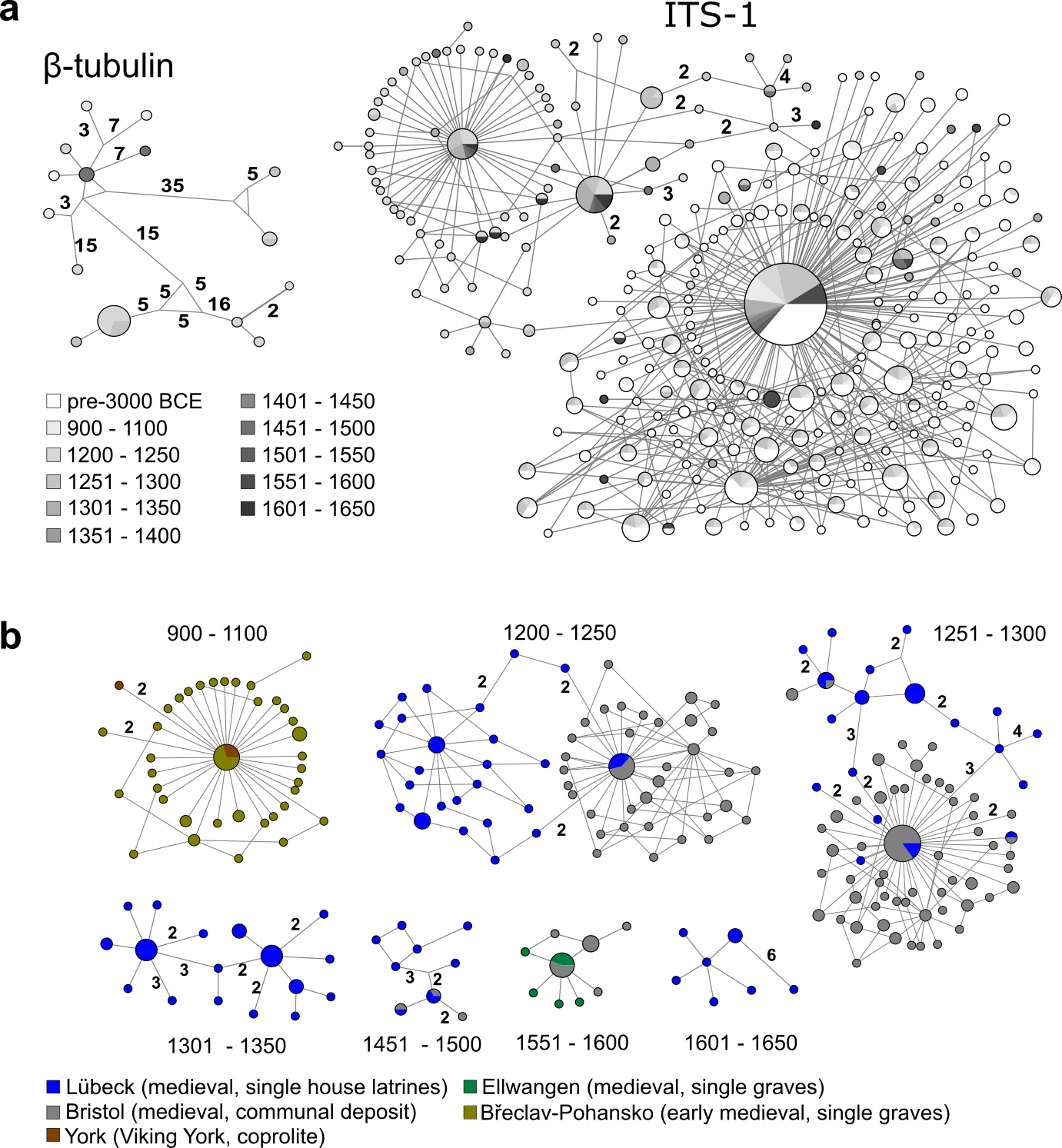


(a) Median-joining haplotype networks, for each gene fragment and coloured by time periods.

(b) Median-joining haplotype networks divided by time period with nodes coloured by site.

The sub-networks visualise the connection between samples from different locations in restricted time periods emphasising the link between Lübeck and Bristol in the early stages of the samples obtained (until 1300 CE).

**Supplementary Table S1: Transition/transversion ratios as a marker for damage in aDNA**

| Species | Target | Ancient DNA | | | | GenBank sequences | | | |
| --- | --- | --- | --- | --- | --- | --- | --- | --- | --- |
| Parasites |  | Ts^a^ | Tv^b^ | n^c^ | Ts:Tv^d^ | Ts^a^ | Tv^b^ | n^c^ | Ts:Tv^d^ |
| *Ascaris* sp. | COX1 | 800 | 57 | 6443 | 14.0:1 | 80 | 21 | 110 | 3.8:1 |
| *T. trichiura* | ITS1 | 79711 | 7748 | 61110 | 10.3:1 | 14 | 3 | 25 | 4.7:1 |
| Putative Food Species | |  |  |  |  |  |  |  |  |
| *Abramis* spp. | mt-16S | 26 | 3 | 182 | 8.7:1 | 2 | 0 | 6 | n/a |
| *Anser* spp. | mt-16S | 1718 | 90 | 1736 | 15.3:1 | 0 | 0 | 20 | n/a |
| *Bos* spp. | mt-16S | 507 | 120 | 5082 | 4.2:1 | 13 | 14 | 250 | 1:1.1 |
| *Clupea* spp. | mt-16S | 816 | 100 | 8219 | 8.2:1 | 18 | 27 | 117 | 1:1.5 |
| *Gadus* spp. | mt-16S | 125 | 6 | 279 | 20.8:1 | 0 | 0 | 50 | n/a |
| *Gallus* spp. | mt-16S | 9273 | 2532 | 110392 | 3.7:1 | 4 | 0 | 108 | n/a |
| *Ovis* spp. | mt-16S | 89 | 14 | 491 | 6.4:1 | 7 | 2 | 120 | 3.5:1 |
| *Sus* spp. | mt-16S | 1425 | 66 | 3004 | 21.6:1 | 4 | 4 | 338 | 1:1 |

^a^Transitions

^b^Transversions

^c^Number of sequences used in the analysis

^d^Ratio of Transitions to transversions

Interpretation of data in Table S1

Most current literature assesses the degradation status of fragmented aDNA sequenced by shotgun sequencing and describe an increase in transition events, principally occurring in the first ~20 bp of the sequence e.g. (1). For PCR amplicons a lower ratio of transition/transversion (Ts:Tv) rates is evident since primers correct many of the end-associated changes and may be positioned at variable distances from the fragment end. However, an increase in Ts:Tv ratio is also reported in PCR amplicons (2). As supporting evidence for aDNA origin of sequences generated in this study we compared the Ts:Tv rates (based on SNP from consensus sequence) for various target sequences reported in this manuscript with those calculated from the equivalent modern sequences available from GenBank (results given in Supplementary Tables S1 and S2). In all cases the Ts:Tv ratios were higher for the aDNA samples than for modern sequences.

Accession number of modern sequences from Supplementary Table 1 are given below:

*Ascaris* sp. (110 sequences)

AB591795-AB591805, AJ968324-AJ968343, AP017677, EU582497, EU628687, EU628688, GU326948-GU326955, HM602025, HQ704900, HQ704901, JN575625-JN801161, KC455923-KC455935, KC839986, KC839987, KF536859, KF536860, KF536865, KF536867, KF536868, KF536871, KF719103, KF719112, KF719113, KF719119, KF719122-KF719124, KF719126, KF719127, KF719130, KF719135, KF719138, KF719142, KF719144-KF719151, KM365020, KM365021, KM365023, KX022397, KY045800, KY045802-KY045805, NC_001327, NC_016198, X54253

*Trichuris trichiura* (25 sequences)

AM992982, AM992983, AM992984, AM992985, AM992986, AM992989, AM992990, AM992991, AM992992, AM992993, AM992994, AM992995, AM992996, AM992997, AM992998, GQ352547, GQ352548, GQ352549, GQ352550, GQ352551, GQ352552, GQ352553, GQ352554, GQ352555, KC877992

*Abramis* spp. (6 sequences)

AJ247067, AP009305, KC894466, KR476906, KR476824, NC_020356

*Anser* spp. (20 sequences)

AF363031, EU932689, HQ890328, KJ124555, KJ778677, KJ794188, KJ794189, KM455570, KP026178, KP238480, KP881611, KP943133, KT427463, KU211647, KY626009, NC_004539, NC_011196, NC_016922, NC_023832, NC_025654

*Bos* spp. (250 sequences)

AB074962-AB074968, AB090988-AB090993, AB098841-AB098849, AB098851- AB098852, AB098854-AB098855, AB098858-AB098864, AB098867-AB098873, AB098875, AB098877-AB098879, AB098881, AB098883-AB098884, AB099098-AB099100, AB099107-AB099109, AB099112, AB099114, AB099117, AB099118, AB099120, AB099122, AB099123, AB099125-AB099129, AB099132, AB099133, AB099137, AB099138, AB099140-AB099142, AB099144, AB099146-AB099148, AB099150, AB511048, AB511049, AF492351, AY236425, AY526085, AY676855-AY676873, FJ002311, GQ129207, GQ129208, GU198195, HM045018, HQ025805, HQ623639, JN817300, JN817301, JN817306-JN817351, KC153972-KC153977, KC561809, KC561810, KC984214, KF163061, KF163094, KF799979, KF926377, KJ709681-KJ709686, KJ789953, KT033901, KT238897, KT238898, KT343748, KT343749, KT375460, KT375462, KT375465, KT375468, KT375469, KT375473, KT375475, KT375482, KT375486, KT375488, KT375491, KT375505, KT375511, KT375517, KT375518, KT375522, KT375529, KT375531, KT375533, KT375538, KT375544, KT375550, KT375558, KT827187, KT827189, KT827190, KT827191, KT827192, KT827195, KT827203, KT827207, KT827208, KT827210, KT827211, KT827216, KT827217, KU891849, KU891850, KX018721, KX018722, KX018723

*Clupea* spp. (117 sequences)

AF420453, AM911204, AP009133, DQ912078, EU552765, EU552766, EU552767, EU552768, EU552769, FN687949, FN687950, GU324147, HQ592201-HQ592203, KC193680-KC193777, KJ128740, KJ128741, NC_009577,X99191

*Gadus* spp. (50 sequences)

AF420462, AM489716, AY141399, AY850363, DQ356939, EU877710-EU877741, FN687991-FN687995, GU018116, GU324163, GU931786, HG514359, KJ128772, KJ128773, NC_002081, X99772

*Gallus* spp. (108 sequences)

AB086102, AP003317-AP003319, AP003321-AP003323, AP003580, AY235570, AY235571, DQ648776, DQ867016, GU261674-GU261719, HQ857209-HQ857212, JQ627250, KF826490, KF908855, KF939304, KF954727, KF981434, KJ778617, KM096864, KM433666, KM886936, KM886937, P211418-KP211425, KP244335, KP269069, KP681580, KP681581, KP742951, KR347464, KT283576, KT626847-KT626858, KT958484, KX512321, KX781318, KX781319, KX987152, KY054997, KY094500, NC_001323

*Ovis* spp. (120 sequences)

AF010406, AY858379, DQ867013, HE577847-HE577849, HE577850, HM236174-HM236185, JX910249, JX910250, KF302440-KF302462, KF938317-KF938359, KF977845-KF977847, KJ954145, KP702285, KP981378-KP981380, KP998470-KP998473, KR814832, KR868678, KT148968, KU575248, KU686952-KU686962, MF004242-MF004246, MF004243, MF004244, MF004245, MF004246, NC_001941

*Sus* spp. (338 sequences)

AB292606, AB298688, AF034253, AF107224, AF304200-AF304203, AF486855-AF486874, AJ002189, AP003428, AY243486-AY243488, AY334492, AY337045, AY574045, AY574045-AY574048, AY920910-AY920914, DQ207753-DQ207755, DQ268530, DQ274110, DQ334810, DQ334860, DQ334861, DQ466081, DQ518915, DQ534707, DQ867012, DQ904382, DQ972936, EF375877, EF545567-EF545593, EU090702-EU090703, EU107789, EU117375, EU333163, FJ236991-FJ237003, GQ220328, GQ220329, GQ338944-GQ338947, GQ351599, GU147934, JF810353-JF810372, JN601066-JN601075, JN714131-JN714133, JN714137-JN714139, JN714149, JN714152-JN714160, KC208030, KC250273-KC250275, KC469586, KC469587, KC493607-KC493612, KC505406-KC505411, KF472177-KF472179, KF569218, KF601700, KF660222, KF752550, KF767443, KF767444, KF799977, KF888634, KF908860, KF926379, KF952600, KF971862, KJ193009, KJ193063, KJ193065, KJ193194, KJ193196-KJ193198, KJ193214, KJ193228, KJ193250, KJ193252-KJ193254, KJ720205, KJ737417-KJ737423, KJ746662-KJ746666, KJ782448, KJ789952, KJ909516, KM044239, KM044240, KM073256, KM094194, KM101042, KM101043, KM200762, KM203891, KM250424, KM259826, KM275217, KM433673, KM998967, KP126939, KP126954, KP223728, KP257598, KP257599, KP294522, KP301137, KP681242-KP681245, KP765602-KP765605, KP789021, KT194217-KT194220, KT238901, KT238902, KT261429, KT261430, KT279758-KT279760, KT316288-KT316291, KT372134, KT827202, KT827206, KT827212, KT827214, KT827218, KT943507, KT965278, KU556691, KX094894, KX146493, KX147100, KX147101, KX620008-KX620012, KX982629-KX982660, NC_000845, NC_012095, NC_014692, NC_023536, NC_023541, NC_023541, NC_024860, NC_026992

**Supplementary Table S2: Site-specific transition/transversion ratios for *T. trichiura* as a marker for damage**

|  | Average Ts:Tv^1^ ratio | Minimum Ts:Tv ratio | Maximum Ts:Tv ratio | | Transition Rate (Tr)  C-T , G-A^2^ | Tr ratio aDNA to modern | TsA:TsB ratio |
| --- | --- | --- | --- | --- | --- | --- | --- |
| Lübeck | 8.38 | 5.8 | 14.3 | | 9.451 10^-3^ | 5.4:1 | 3.894:1 |
| Bristol | 6.83 | 5.1 | 8.7 | | 3.598 10^-3^ | 2.2:1 | 0.171:1 |
| Zurich | 9.37 | 5.4 | 12.5 | | 3.105 10^-3^ | 1.8:1 | 0.599:1 |
| Ellwangen | 5.25 | 5.0 | | 5.5 | 2.358 10^-3^ | 1.3:1 | 0.250:1 |
| Pohansko | 5.73 | 5.1 | 6.2 | | 4.252 10^-3^ | 2.4:1 | 0.520:1 |
| York | 5.40 | Single sample | | | 8.006 10^-3^ | 4.5:1 | 1.610:1 |
| Modern | 4.25^4^ | 3.0^5^ | 5.5^5^ | | 1.766 10^-3^ |  | 1:1 |

^1^ Ts:Tv = Transition:Transversion ratio

^2^ Transition rate calculated as the number of C-T or G-A transitions in unique sequences per source base divided by the number of unique sequences within a sample. For all sites the ratio was higher than in the modern reference samples.

^3^ TsA: Transitions C-T/G-A, TsB: Transitions T-C/A-G

^4^ The ratios are different to supplementary Table 1 as these represent an average of ratios for multiple samples within a site. Note that there were only two sites (at a country level) available for modern data, China and Thailand. Ratios were calculated to the consensus sequence within any individual sample. To avoid inclusion of population structure-defining polymorphisms those represented by >1% were excluded and to avoid sequencing errors we only considered sequences with > 5 repeats.

^5^ These ratios are tentative since only two country-level sites were available, China (15 sequences) and Thailand (9 sequences) with no frequency data. GenBank accession numbers: China: AM992982, AM992983, AM992984, AM992985, AM992986, AM992989, AM992990, AM992991, AM992992, AM992993, AM992994, AM992995, AM992996, AM992997, AM992998; Thailand, GQ352547, GQ352548, GQ352549, GQ352550, GQ352551, GQ352552, GQ352553, GQ352554, GQ352555,).

Interpretation of data in Table S2

Most current literature assesses the degradation status of fragmented aDNA sequenced by shotgun sequencing and describe an increase in transition events, principally occurring in the first ~20 bp of the sequence e.g. (1). For PCR amplicons a lower ratio of transition/transversion (Ts:Tv) rates is evident since primers correct many of the end-associated changes and may be positioned at variable distances from the fragment end. However, an increase in Ts:Tv ratio is also reported in PCR amplicons (2). As supporting evidence for aDNA origin of sequences generated in this study we compared the Ts:Tv rates (based on SNP from consensus sequence) for various target sequences reported in this manuscript with those calculated from the equivalent modern sequences available from GenBank (results given in Supplementary Tables S1 and S2). In all cases the Ts:Tv ratios were higher for the aDNA samples than for modern sequences. The overall Ts:Tv ratios for all targets and sites were greater than those generated from the modern sequences. However, although the TsA:TsB ratio was higher than with modern samples for Lubeck and York the ratio was lower with other archaeological sites. This suggests that at least some of the sequence variation within the PCR products from our samples was attributable to standing population diversity rather than being dominated by aDNA damage.

**Supplementary Table S3: Sequence level identification of potential animal foodstuffs based on mitochondrial 16S sequencing**

|  |  |  | **Proportion of each animal food aDNA sequence (mean%±SEM)^*^** | |
| --- | --- | --- | --- | --- |
|  | **Subfamily/Genus** | **Common name(s)** | **Lübeck** | **Bristol** |
| **Mammals** | *Bos* | Cattle | 7.07±2.75% | 8.76±4.12% |
|  | *Sus* | Pig | 4.07±1.93% | 6.29±4.15% |
|  | Caprinae | Goat/Sheep | 1.21±0.42% | not detected |
|  | *Equus* | Horse | 0.001%^1^ | not detected |
|  |  |  |  |  |
| **Birds** | *Anas* | Duck | 0.015±0.008% | not detected |
|  | *Anser* | Goose | 7.93±2.67% | 0.002%^1^ |
|  | *Gallus* | Chicken | 56.02±5.61% | 84.64±5.57% |
|  |  |  |  |  |
| **Freshwater Fish** | Leuciscinae^2^ | e.g. dace (Leuciscus) | 1.56±1.03% | 0.001%^1^ |
|  | Cyprininae^3^ | e.g. carp (Cyprinus) | 1.31±0.70% | not detected |
|  | *Perca* | Perch | 0.36±0.25% | not detected |
|  | *Lota* | Burbot | 0.05%^1^ | not detected |
|  | *Esox* | Pike | 0.11±0.05% | not detected |
|  | *Coregonus* | Whitefish | 1.48±1.15% | not detected |
|  | *Anguilla* | freshwater eel | 0.57±0.45% | not detected |
|  |  |  |  |  |
| **Marine Fish** | *Clupea* | Herring | 10.90±4.12% | not detected |
|  | *Gadus* | Cod | 3.34±2.28% | not detected |
|  | *Psetta* | Turbot | 0.002±0.001% | not detected |
|  | *Hippoglossus* | Halibut | 0.19±0.12% | not detected |

^1^Sequences only found in one sample

^2^ Subfamily of Cyprinidae, containing *Leuciscus* (dace), *Rutillus* (roach), *Phoxinus* (minnow), *Scardinius* (rudd), *Squalius* (chub) and others

^3^ Subfamily of Cyprinidae, containing *Cyprinus* (common carp), *Abramis* (bream), *Carassius* (crucian carp) and others

^*^Sequencing based upon a mitochondrial 16S fragment (3). The pan-vertebrate approach was possible due to a high-level sequence conservation, which did not segregate species within Cyprinid genera (subfamilies Leuciscinae and Cyprininae) or the ruminant subfamily (Caprinae, likely sheep and/or goat). The percentage signal was calculated with reference to all likely food species aDNA detected (excluding humans, dogs, cats and rodents). A large proportion of sequences in both sites were of human origin (95.21 % in Lübeck and 83.68 % in Bristol).

**Supplementary Table S4:** Nucleotide and relative nucleotide diversities estimated within each location, pairwise *Φ_ST_* among locations and measure of the phylogenetic signal associated with the different geographic origins. (*) indicates significant *p*-values.

|  | Nucleotide diversity  (Nei & Li 1979) | Relative nucleotide diversity  (Mardulyn *et al*. 2009) | Pairwise *Φ_ST_* (Excoffier *et al*. 1992) | | | | |
| --- | --- | --- | --- | --- | --- | --- | --- |
|  |  |  | Lübeck | Bristol | Zurich | York | Ellwangen |
| Lübeck | 0.0130 | 1.7684 |  |  |  |  |  |
| Bristol | 0.0074 | 0.5836 | 0.463* |  |  |  |  |
| Zurich | 0.0076 | 0.5544 | 0.499* | 0.001 |  |  |  |
| York | 0.0034 | 0.2888 | 0.382* | -0.038 | -0.036 |  |  |
| Ellwangen | 0.0029 | 0.2475 | 0.404* | -0.003 | 0.000 | 0.000 |  |
| Pohansko | 0.0064 | 0.5403 | 0.440* | 0.003 | 0.003 | -0.047 | -0.015 |

**Supplementary Table S5:** Parasite sequencing depth and proportion of identified sequence reads

| **Site** | **Target** | **Raw reads** | **Target-specific reads** | **Number of target-specific reads with n>10 replicates/ sample** | **Number of unique sequences (n>10 replicates/ sample)** |
| --- | --- | --- | --- | --- | --- |
| **Lübeck** | *Ascaris* COX1 | 44855 | 5382 | 5103 | 98 |
| **Bristol** | *Ascaris* COX1 | 23072 | 2335 | 2005 | 52 |
| **Ellwangen** | *Ascaris* COX1 | 0 | 0 | 0 | 0 |
| **Pohansko** | *Ascaris* COX1 | 5778 | 857 | 819 | 10 |
| **York** | *Ascaris* COX1 | 201 | 23 | 0 | 0 |
| **Zurich** | *Ascaris* COX1 | 3565 | 256 | 159 | 8 |
| **Lübeck** | *Trichuris* ITS1 | 62536 | 22059 | 15871 | 206 |
| **Bristol** | *Trichuris* ITS1 | 119636 | 36069 | 13738 | 176 |
| **Ellwangen** | *Trichuris* ITS1 | 2795 | 74 | 17 | 1 |
| **Pohansko** | *Trichuris* ITS1 | 42504 | 7703 | 3763 | 55 |
| **York** | *Trichuris* ITS1 | 408 | 139 | 22 | 2 |
| **Zurich** | *Trichuris* ITS1 | 186586 | 54597 | 27699 | 304 |

**Supplementary Table S6:** Vertebrate 16S sequencing depth and proportion of identified reads

| **Site** | **Target** | **Raw reads** | **Target-specific reads** | **Non-food reads** | **[%] non-food^1^** | **[%] of human reads** |
| --- | --- | --- | --- | --- | --- | --- |
| **Lübeck** | Vertebrate 16S | 7522213 | 1128332 | 1074540 | 95.23% | 95.21% |
| **Bristol** | Vertebrate 16S | 3089893 | 313484 | 268684 | 84.71% | 83.68% |
| **Ellwangen** | Vertebrate 16S | 408753 | 35813 | 34887 | 97.41% | 96.84% |
| **Pohansko** | Vertebrate 16S | 1697847 | 209677 | 163210 | 77.84% | 77.77% |
| **Zurich** | Vertebrate 16S | 742173 | 81326 | 80498 | 98.98% | 98.98% |

^1^ Sequences classified as non-food included human, mouse, dogs and rats.

**Supplementary Table S7:** List of reference sequences used for building trees in Figure 2

Figure 2a (*Trichuris trichiura*):

*T. trichiura* (90 sequences): AM992981-AM992998, GQ301554-GQ301555, GQ352547-GQ352555, KC877992, KJ588073-KJ588132

Other *Trichuris* spp.: AM992999-AM993016 (*T. suis*), GQ352556-GQ352558, AM234616 (*T. vulpis*), FN543136, FN543145, FN543171, FN5543194 (*T. muris*)

*Ascaris* spp.: JN176652-JN176654, JN176661, JN176669, AJ554043

Fig 2b (*Ascaris* sp.):

*Ascaris* sp. (205 sequences): AB591795-AB591805, AF182299, AJ968324-AJ968343, AP017677, EF398149, EU582484-EU582499, EU628687-EU628688, GU326948-GU326964, HM602025, HQ704900, HQ704901, JN575625-JN575633, JN801161, KC455923-KC455935, KC839986-KC839987, KC998819-KC998824, KF536859-KF536872, KF719094-KF719151, KM365020, KM365021, KM365023, KT282006-KT282029, KX022397, KY045800-KY045805, NC16198, X54253

Ascaridians: KC293946 (*Toxascaris* sp.), KM216984, EU740387, KJ587840 (*Baylisascaris* sp.)

*Trichuris* spp.: HE653133 (*T. muris*), FR851289 (*Trichuris* sp.), FR851282 (*T. arvicolae*), HE653138 (*T. vulpis*), HE653117 (*T. trichiura*), HE653128 (*T. suis*), HE653143 (*T. ovis*), HE653141 (*T. discolor*)

Fig. 2c (*Taenia* sp.):

*Taenia saginata* (4 sequences): AB271696, AB274525, AB066581, AY684274

*Taenia solium* (15 sequences): JQ973073-JQ973081, AB066574-AB066579, AY211881

*Diphyllobothrium latum*: AB269325, AB522608-AB522612, AP017663, DQ985706

Fig. 2d (*Diphyllobothrium latum*):

*D. latum* (18 sequences): AB302389, AB504899, AB510496, AB511963, AM712906, AM778554, DQ768197-DQ768206, FM209180-FM209181

*Taenia saginata*: AB465233, AB465244, AB465247-AB465248, JN986708-JN986710

*Taenia solium*: AB066490, AB271234, AB524781-AB524785, AB781360

All ancient DNA sequences have been deposited on the NCBI GenBank, accessible under the accession numbers MH599138 - MH600059.

**Supplementary Methods**

**Sample handling and preparation workflow.**

Ancient DNA handling practices have been outlined in a range of publications (4-6). A specialised workflow was developed to prevent any contamination of samples or aDNA by aDNA extracts, PCR amplicons or unprocessed samples. None of the parasites targeted in this study are endemic in the UK or any country where material was received from. None of the laboratories where samples have been processed have ever handled or stored modern samples containing these parasites, hence modern contamination is extremely unlikely.

The workflow was strictly unidirectional for both material and researchers with no equipment or consumables transferred back between steps or the three physically separate locations where work was undertaken. The laboratory where archaeological samples were initially handled and where aDNA was extracted was an extraction clean laboratory (no PCR, no large quantities of DNA handled). The processing was further confined to a dedicated still air hood (UV2 PCR, Ultra-Violet Products Ltd, Cambridge, UK). The workspace in the still air hood was treated to remove contaminants using a combination of UV irradiation (2 x 30min) and ChemGene HLD4L (Medimark Scientific Ltd, Sevenoaks, UK) or 1% Virkon (DuPont UK, Stevenage, UK). Decontamination was performed prior to and after any handling of samples. All PCR was performed in a physically separate, dedicated laboratory space. Where PCR re-amplification was performed these were set up in another dedicated hood (in a second laboratory) decontaminated as described above. All agarose gel analyses and further processing of products occurred in a dedicated space within a third laboratory.

**aDNA extraction.**

Soil subsamples (5 g) were re-hydrated in 20 ml of PCR grade water (Qiagen, Hilden, Germany) as described above. The samples were subsequently filtered through three disposable, single-use nylon mesh sieves. The mesh sizes were 1030 µm, 500 µm and 100 µm (Plastok Associates Ltd, Birkenhead, UK). The sample used for the extraction was the liquid passing through the filters, as the egg sizes are <100 µm and thus would pass all filters while other debris would be retained and disposed of. The flow through was centrifuged (at 400xg 10 min) and the pellet re-suspended in 2ml of Tris-HCl buffer. The samples were homogenised in a BeadBeater (BSP BioSpec, Bartlesville, USA). aDNA was extracted using Qiagen Blood&Tissue or Mericon Food kit (Qiagen, Hilden, Germany).

All PCR reactions contained a “no sample” negative control for each combination of primers (at least one per PCR plate). Blank extraction controls were also performed alongside extractions. Negative controls were included in the sequencing reactions at equivalent proportions to positive samples (as there were no bands on gels and thus no measurable DNA an average amount of PCR product was included). These controls did not yield any sequences. Replicate extractions for selected samples were performed and included in the analysis.

**PCR amplification and sequencing.**

Primary PCR amplification was performed using Invitrogen AmpliTaq Gold 360 (ThermoFisher Scientific, Loughborough, UK) and Phusion Hot Start Flex (New England Biolabs Ltd, Hitchin, UK). The PCR was performed in two stages; with the first boosting the starting concentration using a very robust polymerase (AmpliTaq) and a second stage to produce sufficient amounts of target DNA using a high-fidelity polymerase and to attach the barcodes for MiSeq sequencing (Phusion). Sequencing was run at the Wellcome Trust Centre for Human Genetics (MiSeq) and the Department of Zoology (MiSeq and Sanger), University of Oxford. Primers were designed based on published sequences. Targets were chosen based on commonly used regions or high availability of sequences in public databases. For *T. trichiura* the targets were *β*-*tubulin* (Tri_bT_F: 5’-AGGTTTCAGATACAGTTGTAGAAC-3’, Tri_bT_R: 5’-ATGATTTAAGTCTCCGTAAGTTGGTG-3’, target length 163 bp, primers modified from Diawara (7) and *ITS1* (internal transcribed spacer 1; Tri_ITS1_F: 5’-GCTCGATCAGGCAGCAGCGG-3’, Tri_ITS1_R: 5’-ATTCACGTCAATTCTCGAGTG-3’; target length 185bp). To improve the specificity of the ITS1 sequences, nested primers were designed for the second stage of the PCR (Tri_ITS1_F-N: 5’-GCTCGATCAGGCAGCAGCGGTT-3’). For *Ascaris* sp. *COX1* (Cytochrome c oxidase subunit I) was used, nested primers were designed to improve the accuracy (Asc_A2_F2: 5’-ACTCTCGGGCTTATTTTACTGC-3’, Asc_A2_F2n_p2: 5’-CTCTCGGGCTTATTTTACTGCTG-3’, Asc_A2_R: 5’-ACATAATGAAAATGACTAACAAC-3’, Asc_A2_Rn_p2: 5’-ACATAATGAAAATGACTAACAACTA-3’, target length 241 bp). To identify the cestode identities primers for *T. saginata, T. solium* and *D. latum* were designed. The primers for *T. solium* were based on a Mayta (8) generating a 234 bp fragment of the oncosphere-specific protein *Tso31* (Tso31_Fn: 5’-GGTGTCCAACTCATTATACGCTGTG-3’, Tso31_Rn: 5’-GCACTAATGCTAGGCGTCCAGAG -3’). The primers for *T. saginata* were targeting a 221 bp fragment of the *COX1* region (T.saginata_COX1 _F: 5’-TGCGTTTTTTGATCCATTGGGTGG-3’, T.saginata_COX1_FN: 5’-GTTTTTTGATCCATTGGGTGGTGG-3’, T.saginata_COX1_R: 5’-CATATGATGACCCCACACACTTCTCC-3’, T.saginata_COX1_RN: 5’-ATGACCCCACACACTTCTCCCCAAAC-3’). Primers for *D. latum* were targeting a 200 bp fragment of COX1 modified from Wicht et al. (9) (Diphyllobothrium_COX1_F: 5’-GGGGTGTTACGGGTATTATACTC-3’, Diphyllobothrium_COX1_R2: 5-ATACTTATTCAATCTCACACCTG-3’). The identification of possible food species used a fragment of the mitochondrial 16S region which amplifies a wide range of vertebrate sequences (80-125 bp; primers modified from Horreo et al. (3), 16S-HF: 5'-ATAACACGAGAAGACCCT-3', 16S-HRc: 5'-CCCRCGGTCGCCCCAAC-3'). Sequence chromatograms were visualised using BioEdit v7.2.5 (10). Sequences were aligned using MEGA7 (11). MiSeq pair read data was assembled and dereplicated using USEARCH (12).

**Evaluation of PCR inhibition of aDNA extracts**

Part of the aDNA extraction validation process involved a test for PCR inhibition of the extracts. A dilution series of extracts were added to plasmid DNA (without insert, pCR II, Invitrogen) from which a short fragment (241 bp) was amplified using vector primers (M13). Inhibition was assessed by the difference in intensity of bands on the gel compared to the plasmid-only PCR. Little or no inhibition was detected in any sample group and most samples that were parasite egg positive by microscopy were also positive for parasite PCR products therefore this was not considered a problem.

**Temporal distribution of nematode and cestode eggs in Lübeck.**

The correlations between the numbers of the two nematode parasites *Trichuris* and *Ascaris* in Lübeck and Bristol were evaluated using separate Generalised Linear Mixed Models (GLMMs). Preliminary diagnostic plots revealed a leverage point in the Lübeck dataset that might over-influence the correlation and therefore we omitted that point and re-ran the analysis. The Bristol dataset did not fulfil the assumption of homoscedasticity so we use the non-parametric Spearman’s correlation test instead. To assess whether these shifts are best explained by a gradual change over time or by a sudden shift in parasite prevalence (i.e. a ‘structural break’ in the data) we used Akaike’s information criterion (AIC, (13)) to compare a logit model using date as a predictor of parasite status with logit models in which the predictor is whether a sample derives from before or after a sliding breakpoint.

We analysed variation in parasite occurrence for Lübeck using GLMMs with Binomial error distribution. For each parasite, we entered in separate GLMMs ‘pre or post 1300 CE’ as a fixed factor and ‘building’ (geographical identifier) as a random factor. We also analysed variation in parasite counts using GLMM with a Poisson error distribution ‘pre or post 1300’ as a fixed factor, and ‘building’ as a random factor. The significance of the fixed factors was assessed using the likelihood-ratio test on models with and without the fixed factor (14). All models were checked for over- or under-dispersion. In Poisson-distributed data, we added an observation-level random factor in the model whenever over-dispersion was detected (15,16). No under- or over-dispersion was detected for binomially-distributed data. Because the dataset for Bristol had only four samples post-1300 CE, we utilised non-parametric tests: Fisher’s exact test for the difference in occurrence between pre-1300 CE and post-1300 CE; Mann-Whitney test for the difference in counts between pre-1300 CE and post-1300 CE.

**Sequence processing pipeline**

De-replicated, paired MiSeq reads were processed by pre-screening using BLAST selecting those giving an E value lower than 1x10^-10^ to the expected target before being verified by their location within a bootstrapped maximum likelihood phylogenetic tree (that included appropriate outgroups).

**BLAST parameters**

The species identification of the sequenced PCR amplicons was done using BLAST (17) against the NCBI GenBank. As recommended for this task the “megablast” settings were used (18). These settings are optimised for very similar sequences, e.g. for species identification, based on short, high-scoring, ungapped alignments with a minimal length of 28 nucleotides. The E value was used to judge the quality of the match to any database sequence. Hits with E values above 1x10^-10^ were rejected.

**Molecular phylogenetic analysis**

Phylogenetic history was inferred by using the Maximum Likelihood method under on the Tamura-Nei substitution model (19) with 1000 bootstrap repeats. The percentage of bootstrap replicate trees in which the associated taxa clustered together is shown next to the branches. Initial tree(s) for the heuristic search were obtained by applying Neighbour-Joining or BioNJ algorithms to a matrix of pairwise distances estimated using the Maximum Composite Likelihood (MCL) approach, and then selecting the topology with superior log likelihood value. Phylogenetic analysis was performed using MEGA7 (11). To ensure the correct species identification we included the nearest species (sister species, if possible from within the same genus) and slightly further related species (outgroup).

**Genetic diversity and population structure.**

Nucleotide diversity has been defined as the average number of nucleotide differences per site between any two DNA sequences chosen randomly in a given population (20). The relative nucleotide diversity has been defined as the nucleotide diversity within a given population divided by the nucleotide diversity within the group formed by all other populations (21). These two statistics are by definition corrected for differences in sample sizes. The relative nucleotide diversity statistics compares genetic diversity within each population and investigates if one or several particular population(s) are associated with higher diversity, which is potentially related to the origin area of a range expansion (22). We also used SPADS to estimate the pairwise *Φ_ST_* statistics for *ITS1* (23) among the different locations. Statistical significances of *Φ_ST_* statistics were assessed by recalculating them with 10,000 random permutations of the original datasets.

**Supplementary References**

1. Prendergast ME, Buckley M, Crowther A, Frantz L, Eager H, Lebrasseur O, et al. Reconstructing Asian faunal introductions to eastern Africa from multi-proxy biomolecular and archaeological datasets. PLoS One. 2017;12(8):e0182565.

2. Stiller M, Green RE, Ronan M, Simons JF, Du L, He W, et al. Patterns of nucleotide misincorporations during enzymatic amplification and direct large-scale sequencing of ancient DNA. Proc Natl Acad Sci U S A. 2006;103(37):13578-84.

3. Horreo JL, Ardura A, Pola IG, Martinez JL, Garcia-Vazquez E. Universal primers for species authentication of animal foodstuff in a single polymerase chain reaction. J Sci Food Agric. 2013;93(2):354-61.

4. Paabo S, Poinar H, Serre D, Jaenicke-Despres V, Hebler J, Rohland N, et al. Genetic analyses from ancient DNA. Annu Rev Genet. 2004;38:645-79.

5. Cooper A, Poinar HN. Ancient DNA: do it right or not at all. Science. 2000;289(5482):1139.

6. Hofreiter M, Serre D, Poinar HN, Kuch M, Paabo S. Ancient DNA. Nat Rev Genet. 2001;2(5):353-9.

7. Diawara A, Drake LJ, Suswillo RR, Kihara J, Bundy DA, Scott ME, et al. Assays to detect beta-tubulin codon 200 polymorphism in Trichuris trichiura and Ascaris lumbricoides. PLoS Negl Trop Dis. 2009;3(3):e397.

8. Mayta H, Gilman RH, Prendergast E, Castillo JP, Tinoco YO, Garcia HH, et al. Nested PCR for specific diagnosis of Taenia solium taeniasis. J Clin Microbiol. 2008;46(1):286-9.

9. Wicht B, Yanagida T, Scholz T, Ito A, Jimenez JA, Brabec J. Multiplex PCR for Differential Identification of Broad Tapeworms (Cestoda: Diphyllobothrium) Infecting Humans. Journal of Clinical Microbiology. 2010;48(9):3111-6.

10. Hall TA. BioEdit: a user-friendly biological sequence alignment editor and analysis program for Windows 95/98/NT. Nucleic Acid Symposium. 1999;41:95-8.

11. Kumar S, Stecher G, Tamura K. MEGA7: Molecular Evolutionary Genetics Analysis Version 7.0 for Bigger Datasets. Mol Biol Evol. 2016;33(7):1870-4.

12. Edgar RC, Flyvbjerg H. Error filtering, pair assembly and error correction for next-generation sequencing reads. Bioinformatics. 2015;31(21):3476-82.

13. Akaike H. A New Look at the Statistical Model Identification. IEEE Transactions on Automatic Control. 1974;AC-19(6):8.

14. Zuur AF, Ieno EN, Walker N, Saveliev AA, Smith GM, service) SO. Mixed effects models and extensions in ecology with R: SpringerLink Online service; 2009. 1 online resource (XXII, 574p.) p.

15. Harrison XA. Using observation-level random effects to model overdispersion in count data in ecology and evolution. PeerJ. 2014;2:e616.

16. McCullagh P, Nelder JA. Generalized linear models. 2. ed. London u.a.: Chapman & Hall; 1992. XIX, 511 S p.

17. Altschul, S. F., W. Gish, W. Miller, E. W. Myers and D. J. Lipman (1990). "Basic local alignment search tool." J Mol Biol 215(3): 403-410.

18. Zhang, Z., S. Schwartz, L. Wagner and W. Miller (2000). "A greedy algorithm for aligning DNA sequences." J Comput Biol 7(1-2): 203-214.

19. Tamura K, Nei M. Estimation of the number of nucleotide substitutions in the control region of mitochondrial DNA in humans and chimpanzees. Mol Biol Evol. 1993;10(3):512-26.

20. Nei M, Li WH. Mathematical model for studying genetic variation in terms of restriction endonucleases. Proc Natl Acad Sci U S A. 1979;76(10):5269-73.

21. Mardulyn P, Mikhailov YE, Pasteels JM. Testing phylogeographic hypotheses in a Euro-Siberian cold-adapted leaf beetle with coalescent simulations. Evolution. 2009;63(10):2717-29.

22. Dellicour S, Mardulyn P, Hardy OJ, Hardy C, Roberts SP, Vereecken NJ. Inferring the mode of colonization of the rapid range expansion of a solitary bee from multilocus DNA sequence variation. J Evol Biol. 2014;27(1):116-32.

23. Excoffier L, Smouse PE, Quattro JM. Analysis of molecular variance inferred from metric distances among DNA haplotypes: application to human mitochondrial DNA restriction data. Genetics. 1992;131(2):479-91.
